# Optical Properties of MoSe_2_ Monolayer Implanted with Ultra-Low-Energy Cr Ions

**DOI:** 10.1021/acsami.3c05366

**Published:** 2023-07-11

**Authors:** Minh N. Bui, Stefan Rost, Manuel Auge, Lanqing Zhou, Christoph Friedrich, Stefan Blügel, Silvan Kretschmer, Arkady V. Krasheninnikov, Kenji Watanabe, Takashi Taniguchi, Hans C. Hofsäss, Detlev Grützmacher, Beata E. Kardynał

**Affiliations:** † Peter Grünberg Institute 9 (PGI-9), Forschungszentrum Jülich, 52425 Jülich, Germany; ‡ Department of Physics, RWTH Aachen University, 52074 Aachen, Germany; § Peter Grünberg Institute 1 (PGI-1) and Institute for Advanced Simulation 1 (IAS-1), Forschungszentrum Jülich and JARA, 52425 Jülich, Germany; ∥ II. Institute of Physics, University of Göttingen, 37077 Göttingen, Germany; ⊥ Institute of Ion Beam Physics and Materials Research, Helmholtz-Zentrum Dresden-Rossendorf, 01328 Dresden, Germany; # Department of Applied Physics, Aalto University School of Science, P.O. Box 11100, 00076 Aalto, Finland; δ Research Center for Functional Materials, National Institute for Materials Science, 1-1 Namiki, Tsukuba 305-0044, Japan; ° International Center for Materials Nanoarchitectonics, National Institute for Materials Science, 1-1 Namiki, Tsukuba 305-0044, Japan

**Keywords:** transition-metal dichalcogenide monolayer, ultra-low-energy ion implantation, MoSe_2_, van der Waals heterostructure, photoluminescence, molecular dynamics, density functional theory

## Abstract

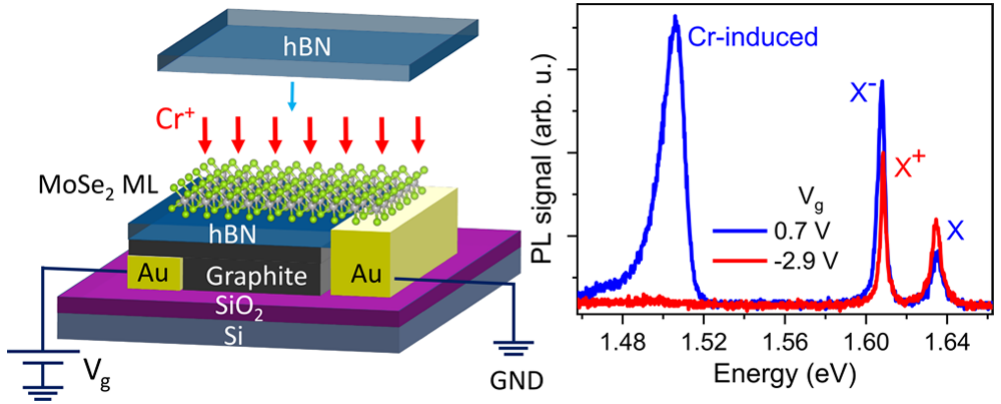

This paper explores the optical properties of an exfoliated MoSe_2_ monolayer implanted with Cr^+^ ions, accelerated to 25 eV. Photoluminescence of the implanted MoSe_2_ reveals an emission line from Cr-related defects that is present only under weak electron doping. Unlike band-to-band transition, the Cr-introduced emission is characterized by nonzero activation energy, long lifetimes, and weak response to the magnetic field. To rationalize the experimental results and get insights into the atomic structure of the defects, we modeled the Cr-ion irradiation process using ab initio molecular dynamics simulations followed by the electronic structure calculations of the system with defects. The experimental and theoretical results suggest that the recombination of electrons on the acceptors, which could be introduced by the Cr implantation-induced defects, with the valence band holes is the most likely origin of the low-energy emission. Our results demonstrate the potential of low-energy ion implantation as a tool to tailor the properties of two-dimensional (2D) materials by doping.

## 1 Introduction

The properties of semiconductors, especially atomically thin monolayer (ML) semiconductors, depend strongly on the types and densities of defects in their crystal lattices. The most technologically relevant defects are dopants, i.e., foreign atoms in substitutional positions in the crystal lattice. Shallow dopants introduce free electrons or holes into the conduction or valence band and thus change the semiconductor conductivity. As such, they facilitate the fabrication of p–n junctions, which underpins most active optoelectronic devices.

Doping with transition-metal atoms has been shown to introduce ferromagnetic order in p-doped semiconductors.^1^ Impurity atoms can also trap electrons or holes or bind excitons. Radiative recombination involving such states can be detected as sub-band-gap photoluminescence (PL). Single foreign atom that bind excitons have been explored for single photon sources.^2^ Alternatively, if the dopant atom has a functionality of a spin qubit, the bound excitons provide an optical readout of its state.^3,4^ The binding of excitons to the dopant atoms depends not only on electron and hole masses but also on the dielectric constant of the semiconductors. Because of that, excitonic effects in bulk semiconductors are only observed at cryogenic temperatures. Foreign atoms can also act as color centers in semiconductors and insulators. Spin qubits based on the color centers have been realized in diamond^5,6^ or SiC.^7,8^

In two-dimensional (2D) semiconducting transition-metal dichalcogenides (TMDs), which feature weak electrostatic screening, substitutional atoms tend to introduce deep levels in the band gaps.^9^ While excitons have considerable binding energies, they are predicted to be very weakly bound to individual doping atoms.^10^ Optical transitions involving defect states have been observed,^11^ with defects identified as vacancies.^11−14^ The transition responsible for the PL was found to occur between the hybridized defect states and 2D lattice electronic states.

Among several methods of doping bulk semiconductors, ion implantation offers the highest flexibility in choosing implanted elements. Ion energies of tens of keV are used for implantation since functional layers can be even a hundred nanometers below the surface. High-energy ion implantation has been used to modify 2D materials,^15−17^ but its efficiency is low in this case, as most atoms go through the 2D target.^18^ Moreover, the ions penetrating through the ML can cause undesirable effects, e.g., trapped charges in the substrate. Implantation into 2D materials has the highest implantation efficiency with ion energies in the range of tens of eV. At these energies, the implantation efficiency and threshold energy depend on the ions’ mass and also chemical properties.^19^ Ultra-low-energy ion implantation^20,21^ has recently been demonstrated to be efficient in doping graphene using 40 eV Mn ions^22,23^ or in Se ion implantation into MoS_2_ with an ion energy of 20 eV.^24,25^ The ratio of the replaced S atoms with Se in the top sublattice was sufficient to form a Janus compound MoS_2–2*x*_Se_2*x*_ as indicated by Raman spectroscopy and the transmission electron microscopy imaging.

Here, we study the optical properties of molybdenum diselenide (MoSe_2_) ML implanted with 25 eV ^52^Cr^+^ ions. Sub-band-gap defect-induced PL emission was observed only at the low n-doping level and with saturation behavior, characteristic of defects with low density. Ab initio molecular dynamics (MD) simulations of the implantation process were performed, and possible configurations of the Cr atoms in the MoSe_2_ ML lattice were outlined to understand the atomic structure of the implanted MLs. The optical properties of the MoSe_2_ ML with such defects were calculated using density functional theory (DFT). The most probable defect configurations were identified by combining experimental data and theoretical calculations.

## 2 Results

### 2.1 Sample Preparation and Ion Implantation

**Figure 1:**
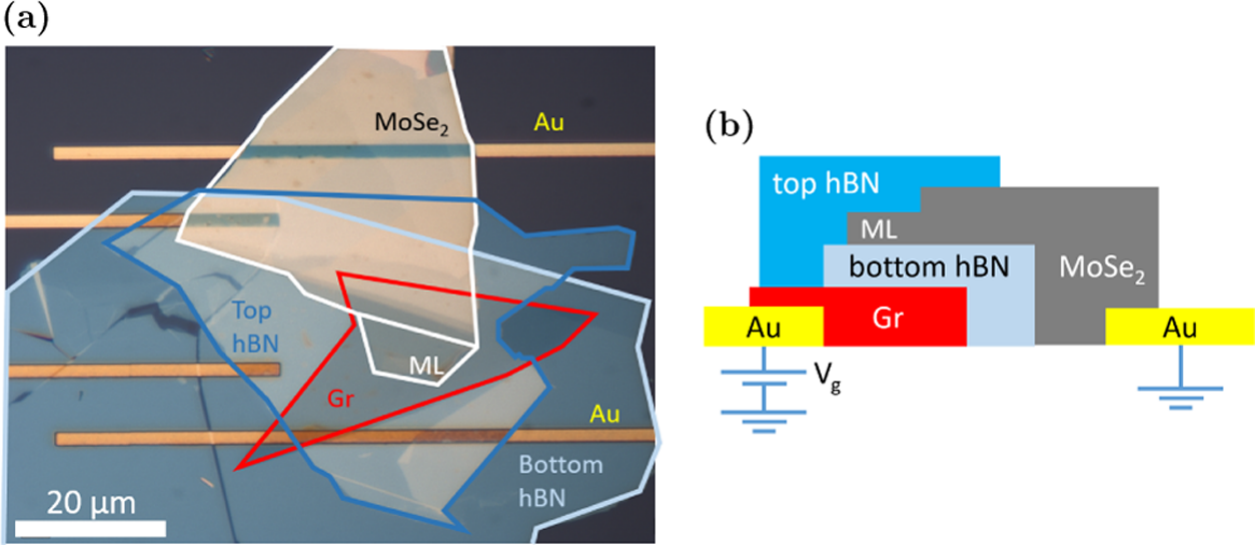
Cr-implanted MoSe_2_ ML with hBN encapsulation and graphite backgate. (a) Micrograph of the finished device. The ML part of the exfoliated MoSe_2_ flake is encapsulated between two thin hBN flakes. The few-layer graphite backgate and the thick part of MoSe_2_ flake make contacts with the two Ti/Au lines to the right. (b) Schematic diagram of the device cross section. Backgate voltage *V*_g_ can be applied to the graphite backgate via the Au contact, while the MoSe_2_ flake is grounded via the other Au contact.

A sample for ion implantation was prepared by mechanical exfoliation of the MoSe_2_, graphite (Gr), and hexagonal boron nitride (hBN) flakes and their sequential transfer onto the Si/SiO_2_ substrate with pre-patterned Ti/Au contacts. The use of the dry viscoelastic transfer technique^26^ ensured that the surface of the ML was sufficiently clean for the implantation. The MoSe_2_ ML has to be grounded during the implantation. An electric contact to the ML was provided by placing the multilayer part of the exfoliated MoSe_2_ flake on a Ti/Au metal contact. The ML was placed atop a graphite gate connected to another Ti/Au contact. The ML was separated from the gate with an hBN flake. Once completed, the device was implanted with ^52^Cr^+^ ions at 25 eV and a fluence of 3 × 10^12^ cm^–2^ (equivalent to 0.003 Cr per ML MoSe_2_ unit cell, using the MoSe_2_ ML in-plane lattice constant 3.32 Å^27^). The implantation was performed with the device heated to 220 °C. Following the implantation and initial characterization, another hBN flake was deposited on the ML MoSe_2_ for full encapsulation, which protects the ML from interactions with the environment during the optical measurement and reduces the inhomogeneous broadening in the PL linewidth.^28^ The device was annealed at 150 °C to improve the interface between the constituent layers.^28^ The complete device is shown in Figure 1. More details are available in Section 5.

### 2.2 Optical Spectroscopy Data

**Figure 2:**
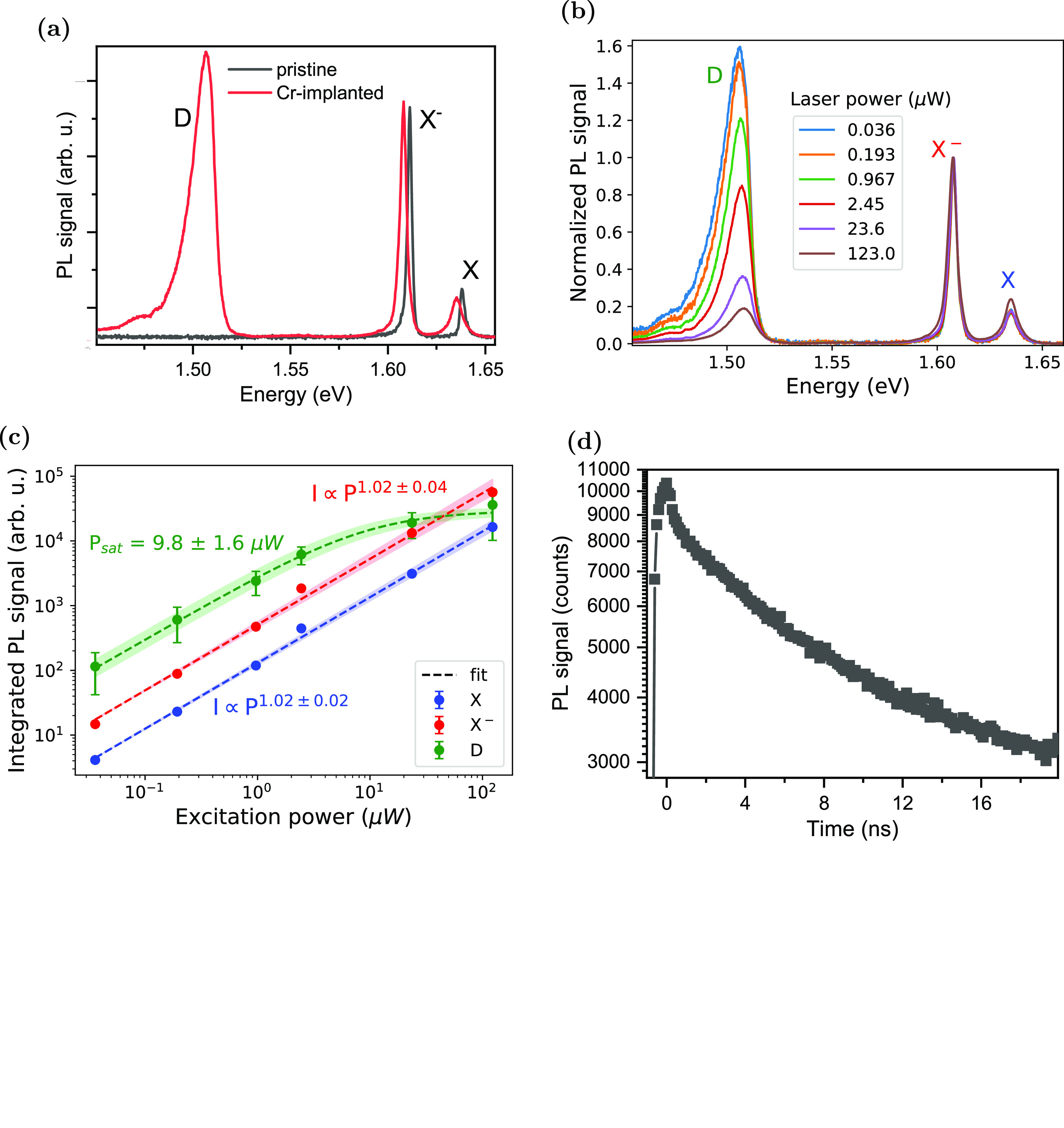
PL of Cr-implanted MoSe_2_ ML at 10 K. (a) PL spectra of Cr-implanted MoSe_2_ ML (red curve) at low n-doping (*V*_g_ = 0.8 V), plotted with that of pristine MoSe_2_ ML (black). In addition to the X^–^ and X from MoSe_2_ ML, the Cr-implanted sample also shows the broad D peak at around 1.51 eV. (b) PL spectra of Cr-implanted ML under laser power ranging from 36 nW to 123 μW. Spectra are normalized to X^–^. Here, the sample is slightly n-doped at *V*_g_ = 0.8 V. (c) Power dependence of PL. Best-fit lines (dashed), with their standard deviations (shaded region around the lines), are plotted together with the extracted intensity from PL spectra (dots). Unless explicitly shown, the error bars are smaller than the size of the data points. X^–^ and X are fitted with power law *I* ∝ *P*^α^, and D is fitted with the saturation curve described by eq 1. (d) Time-resolved PL of Cr-implanted MoSe_2_. 1/*e* time is around 14 ns.

Figure 2a shows PL spectra of weakly electron-doped pristine and Cr-implanted MoSe_2_ MLs, measured at 10 K with the same laser power of 1 μW. Both spectra show similar features around the band-gap transitions, with an emission line from neutral excitons (X) and negative trions (X^–^). The red shift of these transitions in the Cr-implanted sample (Figure 2a) is most likely due to a difference in the dielectric environment or strain between the samples. The level of implantation is too low to expect changes in the band gap.^29^ Significant homogeneous broadening of the X line was determined by the Voigt function fitting. It resulted in the Lorentzian width of nearly 7 meV for the implanted sample, compared to about 2 meV for the pristine sample, suggesting a much shorter lifetime of the former. The most significant difference between the samples is a broad emission at around 1.51 eV, which we label D.

The relative intensity of the D peak compared to X^–^ and X depends on the excitation power *P* (Figure 2b). It is the most intense line at low laser excitation powers (*P* < 1 μW) but saturates as the laser power increases, while X^–^ and X continue to grow linearly. The saturating behavior of the D intensity, shown in Figure 2c, can be expressed phenomenologically as
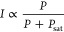
1with saturation power *P*_sat_ ≈ 10 μW. The saturating behavior is expected when the exciton generation rate exceeds the recombination rate of the states responsible for D. The saturation threshold depends on the density of states and the lifetime of the recombining carriers.^11,13^ The low threshold for D is consistent with the low implantation level. The lifetime of carriers was measured from time-resolved PL. The decay of the population of the excited states contributing to the D peak after a pulsed excitation can be seen in Figure 2d. As can be expected from measuring an ensemble of emitters, the decay is not a single exponential. The very fast initial decay of population by about 10%, which is faster than the time resolution of the experiment, is followed by a slower decay with the 1/*e* decay time of around 14 ns. These decay times are 2–3 orders of magnitude longer than the lifetime of free excitons in MoSe_2_ MLs^30,31^ and one order of magnitude longer than that from the isolated, confined excitons,^32^ pointing to a low oscillator strength of the emitters.

**Figure 3:**
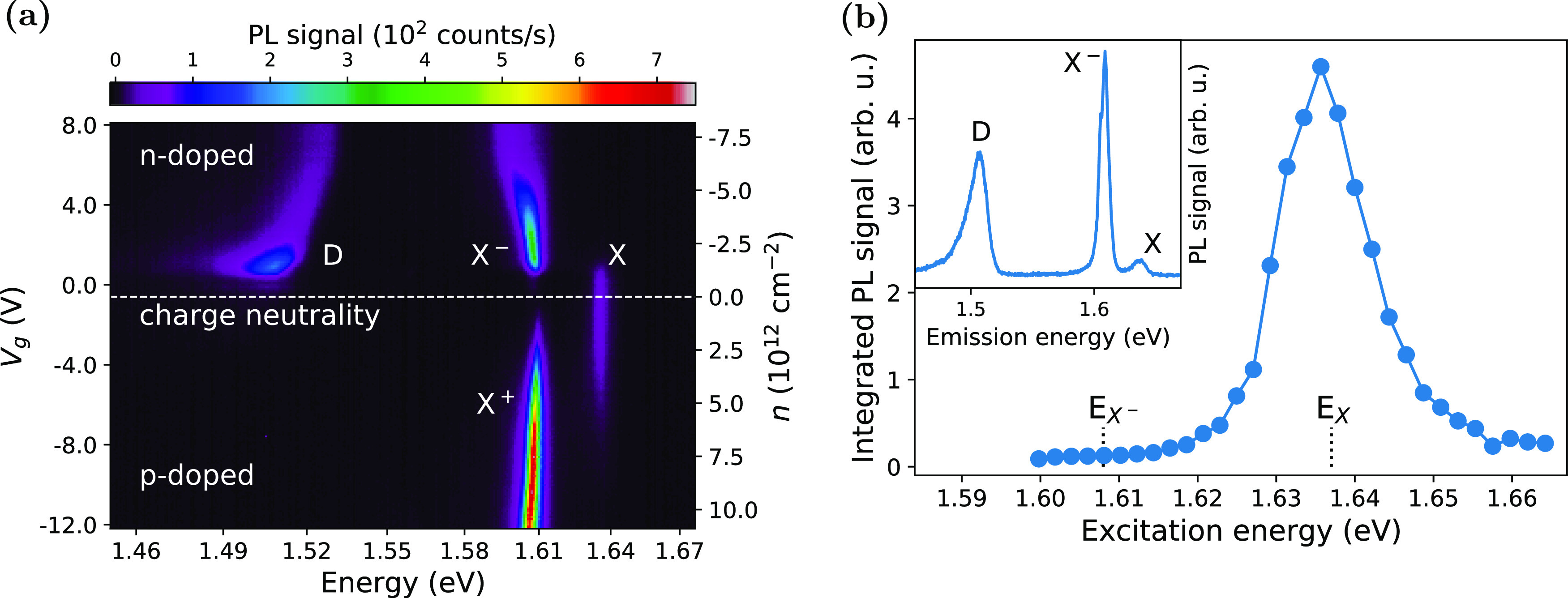
Doping level and excitation energy dependencies of PL. (a) Gate-dependent PL, where the backgate voltage *V*_g_ was varied from −12 to 8 V to tune the doping level in the ML from p- via neutral to n-doping. The carrier concentration *n* is calculated using the simple parallel plate capacitor model (more details in the Supporting Note 7). (b) PLE of the D peak, taken at *V*_g_ = 0.7 V. The D peak intensity was integrated around its PL emission energy between 1.48 and 1.52 eV. Inset: PL spectrum under a 688 nm (1.80 eV) excitation under the gate voltage as applied for the PLE measurements.

The doping-dependent PL from neutral and charged excitons shown in Figure 3a is typical of MoSe_2_ MLs. When increasing the gate voltage, the D emission only emerges after the signal from the positive trion, X^+^, entirely disappears. It reaches the maximum intensity around 1 V; the gate voltage of the transition between X and X^–^ dominated spectra. The blueshift of the D emission at higher gate voltage is likely to be due to the energy renormalization due to the screening by the free charges in the ML. Similar behavior for defect peaks was observed by others.^33^ The D line weakens strongly as the X^–^ line intensifies with a further gate voltage increase. This behavior suggests a competition between the exciton capture at the defect state and the formation of an X^–^. This scenario is supported by the PL excitation (PLE) spectroscopy measurement (Figure 3b), which shows that the intensity of the D emission is maximum when the excitation wavelength is resonant with the energy of X around 1.637 eV. The D emission was not excited with laser resonant with X^–^ (around 1.608 eV) even though the PL spectrum, measured at the same doping level, clearly shows that the ML is doped with electrons (X^–^ emission is the strongest PL signal).

**Figure 4:**
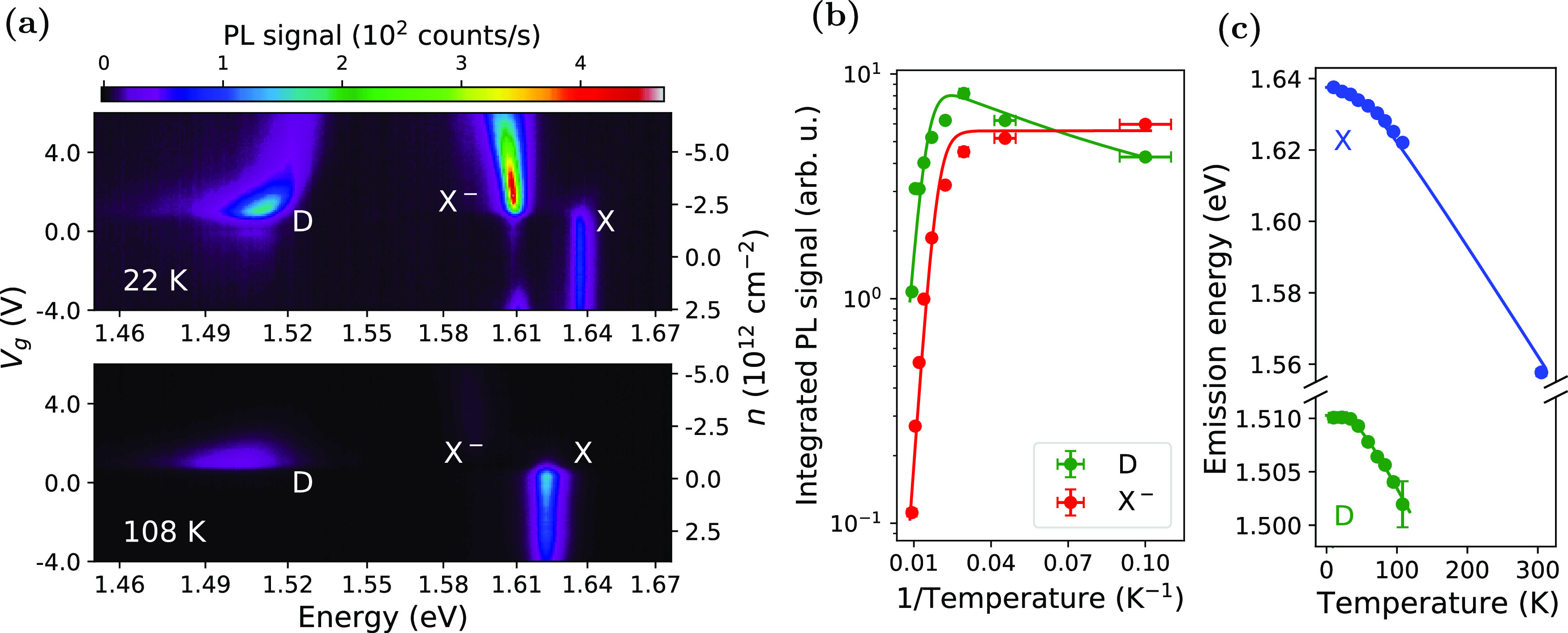
Temperature dependence of PL emission. (a) Gate voltage-dependent PL at 22 and 108 K. (b) Arrhenius plot of X^–^ and D (symbols). At each temperature, the two peaks’ integrated intensities were acquired at the doping levels, where each emission is the brightest. The intensities were then normalized to that of X (at the voltage where X is most intense). The best fit lines were according to eqs 2 and 3. (c) The temperature-dependent band gap of X and D (symbols). The best-fit line was according to eq 4.

Figure 4a compares gate-dependent PL at 22 and 108 K. While X^–^ emission is the most intense line in the spectra at the lower temperature, it is very weak at the higher temperature. X becomes the strongest line, but D diminished less than X^–^ and remains up to room temperature (Supporting Figure S4b). We trace the change of the PL signal counts from X^–^ and D, both normalized to X signal counts, on the Arrhenius plot shown in Figure 4b. X^–^ dissociates into higher-energy X and an electron at a higher temperature. Its intensity can be fitted with the standard Arrhenius formula^34^
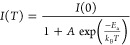
2where *I*(0) is the PL intensity at temperature 0 K, *A* is a proportionality constant, *E*_a_ is the activation energy for the dissociation of X^–^, and *k*_B_ is the Boltzmann constant. Fitting the formula to the data gives *E*_a_ ≈ 32 ± 5 meV, which is expected for a binding energy of the trion.^10,35^ The D emission intensity first increased with temperature up to around 34 K before diminishing. To account for this initial increase in intensity, we assume that the trapping of carriers that recombine requires overcoming the activation energy. We use a modified multilevel model for the temperature dependence of the D intensity^34^
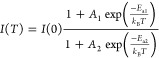
3where *A*_1_ and *A*_2_ are the proportionality constants, and *E*_a1_ and *E*_a2_ are the activation energies for trapping and detrapping of carriers, respectively. Fitting of the D peak yields *E*_a1_ ≈ 1.2 ± 0.8 meV and *E*_a2_ ≈ 30 ± 7 meV.

Temperature affects not only the intensity but also the D emission energy. The temperature-dependent energy shift of X and D lines can be described by the modified Varshni relation^13,36^ as
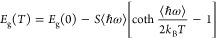
4

where *E*_g_(0) is the emission energy at 0 K, *S* is the electron–phonon coupling, and ⟨ℏω⟩ is the average phonon energy. Fitting gives ⟨ℏω⟩ = 11.1 ± 1.3 meV and *S* = 1.82 ± 0.14 for X, similar to the reported values (⟨ℏω⟩ ≈ 12–20 meV, *S* ≈ 2.^37−39^). For the D emission, the fitted ⟨ℏω⟩ and *S* are 10.7 ± 1.5 and 0.79 ± 0.09 meV, respectively. A smaller *S* constant compared to excitonic lines has been reported for vacancy-induced PL emissions from TMD MLs^13,14,40^ and explained as a result of the defect being decoupled from the conduction band, which varies with the temperature. A similar scenario is also likely to be the case in our sample.

**Figure 5:**
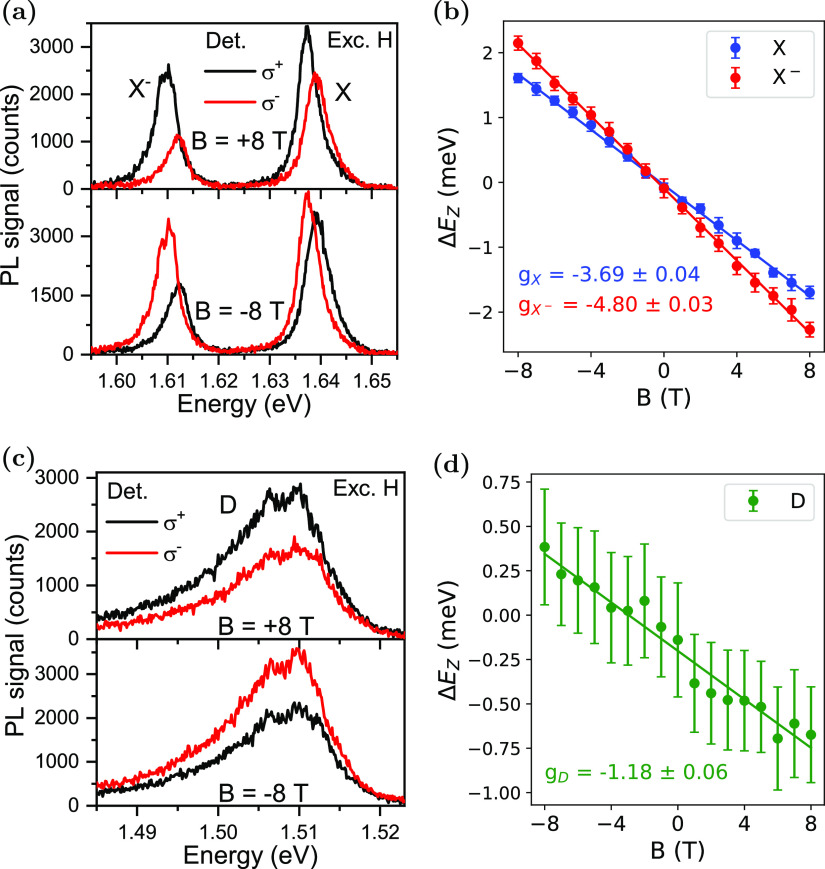
Magneto-PL measurement of Cr-implanted MoSe_2_ ML at 1.8 K. PL spectra acquired with out-of-plane magnetic field *B* (varying between −8 and 8 T) applied to the sample and excited with an H-polarized laser. The detection is set to measure either σ^+^ (black) or σ^–^ (red) polarization states. The figure shows the polarization-resolved PL spectra of (a) X and X^–^, (c) D, with their Zeeman splitting Δ*E*_Z_ shown in (b, d). The splitting was calculated from the peak positions (extracted from fitting Voigt functions to the PL spectra), with the error bars representing the propagated standard deviation of the fit procedure. The Zeeman splitting of the three emissions reveals expected *g*-factors around −4 for X and X^–^, only around −1.18 for D.

To gain further insight, we measured the PL emission from the sample under the out-of-plane magnetic field *B* varying from −8 to 8 T. Figure 5a shows the splitting of X and X^–^ spectra in two circular polarization detection states under the applied *B*-field. The valley splitting, caused by the Zeeman effect^41,42^ and defined as
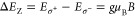
5(where *E*_σ^+^_ and *E*_σ^–^_ are the emission energy in the detected circular polarization basis σ^+^ and σ^–^, respectively, *g* is the Landé *g*-factor, μ_B_ is the Bohr magneton) changes linearly with the applied magnetic field (Figure 5b). The *g*-factors derived from the data are −3.69 ± 0.04 and −4.80 ± 0.03 for X and X^–^, respectively. The *g*-factor value for X is close to the ones from the previous experimental work,^28,41−45^ which are between −3.8 and −4.3 and well within the expected range from −3.22 to −3.82 predicted by recent ab initio calculations.^46,47^ The *g*-factor for X^–^ is slightly higher than for X but similar to the values observed for samples under higher doping level.^43−45^ On the other hand, the D emission shows little change with the magnetic field (Figure 5c). Comparing the energy of photons from the D peak in both circular polarization gives a *g*-factor of about −1.18 ± 0.06. The peak position was determined by fitting the data with three Voigt functions and then taking the maximum of the fitted line. The uncertainty here is high, partly owing to the D peak’s large width.

### 2.3 First-Principles Molecular Dynamics Simulation of Cr-Ion Implantation into MoSe_2_ ML

**Figure 6:**
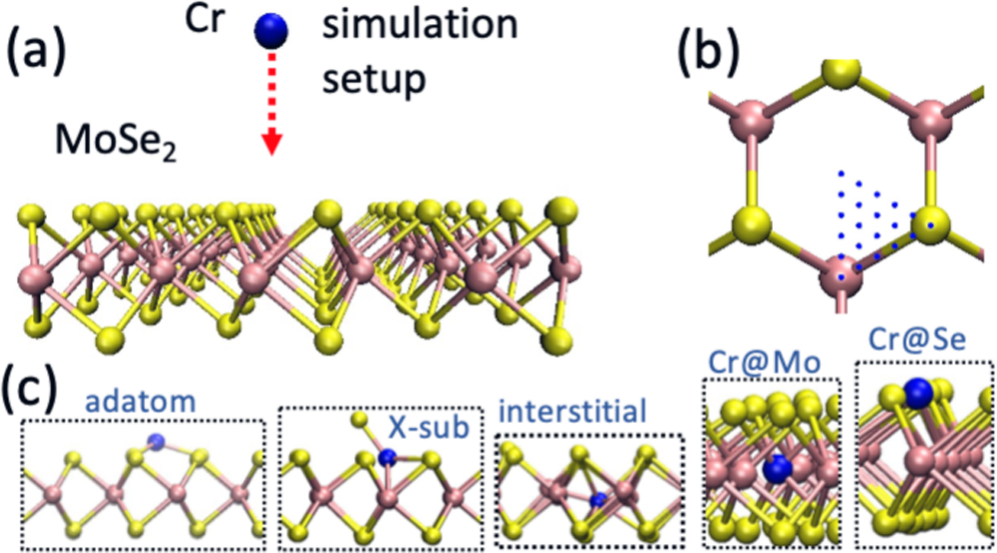
First-principles MD simulation of the ion implantation process into ML MoSe_2_. (a) The setup for simulations of ion impacts. (b) Impact sites used in the simulation. (c) Atomic structures of the defects likely to appear upon impacts of energetic Cr ions.

To get insights into the defect formation process and types of defects that can appear upon impacts of energetic Cr ions, we carried out DFT MD simulations, as described below. The atomic structure of a free-standing MoSe_2_ rectangular slab containing 90 atoms was fully optimized; then, a Cr atom was placed 6 Å above its surface (Figure 6a) and a kinetic energy of 25 eV was assigned to the atom. Normal incidence was simulated; that is, the initial velocity vector of the projectile was oriented perpendicular to the surface of the ML. The projectile was assumed to be a neutral atom, as at such low energies and low charge states, its neutralization must occur well before it reaches the surface. We note that DFT MD on the Born–Oppenheimer surface cannot describe the evolution of charge transfer anyway, and the Ehrenfest dynamics^48,49^ should be used. 21 impact points were selected in the irreducible area of the primitive cell of MoSe_2_ (Figure 6b), and the outcomes of the simulations were averaged with the corresponding weights. The MD runs continued until the kinetic energy brought up by the projectile was distributed over the whole supercell (normally after a few picoseconds); then, the system’s temperature was quenched to zero, and the atomic structure was analyzed. Although the effects of substrate on defect generation in a 2D system can be significant for ions with much higher (keV range) energies,^50−52^ the role of the substrate should be minimal for impacts of 25 eV Cr ions onto MoSe_2_, so that a free-standing slab was simulated. Spin-polarized calculations were carried out. Although computationally more efficient non-spin-polarized method with a correction for isolated atom polarization energies can be used to simulate irradiation effects,^19^ the account for spin effects is particularly important for Cr, as it is magnetic, which affects the energetics of defect configurations.

Figure 6c shows the most common atomic configurations that appear after Cr atom impacts. These are Cr adatoms, X-sub configuration (Cr at Se sites with a Se adatom^53^), interstitials (the Cr atom between Mo atoms), and substitutional defects in Mo and Se sites, which are Cr@Mo and Cr@Se, respectively. Table 1 lists the probabilities for the defects to appear. Ion irradiation also gives rise to the sputtering of Se atoms, that is, the formation of Se vacancies (V_Se_), but these events were not so common.

**Table 1:** Results of DFT MD Simulations of a 25 eV Cr-Ion Irradiation on a Single-Layer MoSe_2_^a^

	*p*	*E*_f_ [eV]
adatom	0.16	–0.85
X-sub	0.41	–0.76
interstitial	0.08	–0.25
Cr@Mo	0.21	3.03
Cr@Se	0.04	2.57
V_Se_	0.01	5.41
passed through	0.09	0.00

a The probabilities *p* of likely defect configurations to appear along with the formation energies *E*_f_ of these configurations are listed.

According to the DFT MD simulations, the most probable defects that appear upon 25 eV Cr-ion irradiation are Cr adatoms, Cr@Mo, and X-sub defects. The Cr atoms that pass through the MoSe_2_ sheet will likely form adatoms attached to the bottom of MoSe_2_. Self-annealing of defects at finite temperatures at which irradiation was carried out in the experiment can affect their concentrations in the implanted samples. To get insight into the possible evolution of defects, we assessed the defect formation energies *E*_f_, as done previously.^53^ For adatoms, interstitials, and X-sub defects, *E*_f_ was calculated as the energy difference between the system with the Cr atom and the pristine system plus isolated Cr atom. For the Cr@Mo, Cr@Se, and V_Se_ configurations, the energies of isolated Mo and Se atoms were also taken as a reference. We note that the listed defect formation energies for the Cr@Mo, Cr@Se, and V_Se_ cannot be used to assess the equilibrium concentrations of these defects, as the chemical potentials were chosen to match isolated, that is, sputtered, atoms. This can be done, though, if the chemical potentials of the displaced Se and Mo atoms are chosen in such a way that they reflect the actual experimental conditions that the potential can be anywhere between the values corresponding to the Se- or Mo-rich limits. This would result in lower formation energies, as the sputtered atoms would be incorporated into the lattice. It can also be assumed that the displaced Se atoms form Se clusters at the surface, which would give rise to the lowering of Cr@Se defect energies.

As evident from Table 1, *E*_f_ for adatoms is lower than for the interstitials, so that at finite temperatures, the interstitials will most likely be “pushed away” from the Mo plane and form adatoms. We note that this result was obtained for a relatively small 90-atom supercell, and in the larger system, the difference between these energies is smaller, as reported earlier.^53^ Nevertheless, even for equal formation energies at zero temperature, with an account for the entropic term in the Gibbs energy, the probabilities for the adatoms should be higher due to a larger configurational space. Some X-sub defects may also be converted to Cr@Se configurations, especially in the Mo-rich limit, when Se vacancies are present, but the energetics of this process naturally depends on the experimental conditions, that is, the choice of Se chemical potential. The Cr@Se defects can also appear due to the adsorption of Cr atoms on Se vacancies, as this is energetically favorable due to the saturation of dangling bonds. Thus, one can expect that the most prolific defects in the samples are Cr adatoms (or Cr clusters on top of MoSe_2_), X-sub, as well as Cr@Mo and Cr@Se substitutional configurations.

### 2.4 DFT Calculations of Optical Properties

**Figure 7:**
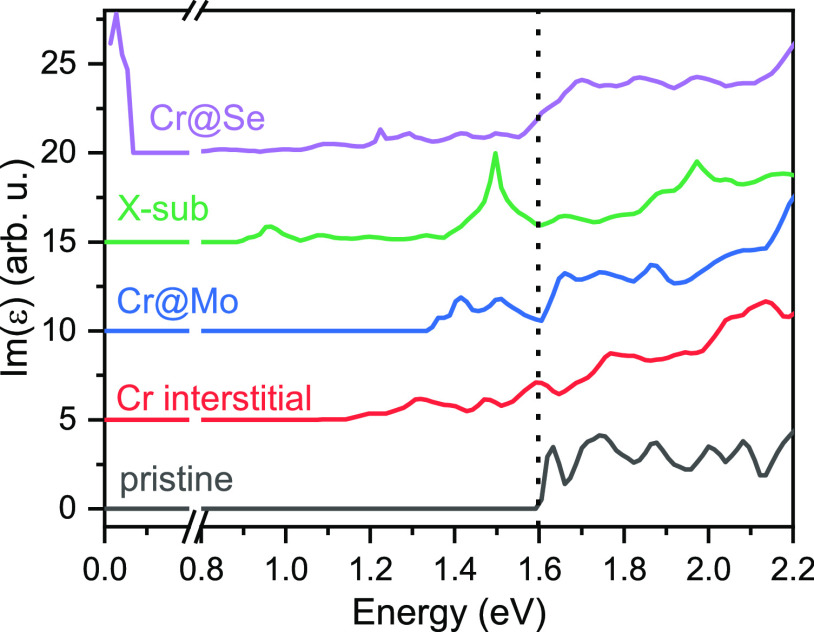
Comparison of calculated absorption functions for pristine MoSe_2_ ML (black line) and MoSe_2_ ML with Cr in various positions of the crystal structure: Cr at the interstitial position (red), Cr at the Mo position (Cr@Mo—blue), Cr at the Se position with additional Se adatom (X-sub, green), and Cr at the Se position (Cr@Se, purple). Spectra are offset vertically for clarity. The vertical dotted line at about 1.6 eV marks the calculated band gap of pristine MoSe_2_ ML.

To investigate if Cr defects introduce states in the band gap of the MoSe_2_ ML that are optically active, we have simulated optical absorption spectra for MoSe_2_ with Cr defects in various positions. The simulations are based on a 5 × 5 supercell. Each supercell hosts one Cr defect. The unfolded band structures are shown in the Supporting Figure S5. All defects give rise to states in the band gap. The absorption spectrum Im[ε(ω)] with the energy-dependent macroscopic dielectric function ε(ω) has been calculated within the random-phase approximation in the limit **k** → **0**. Optical matrix elements and local-field effects are taken into account. It should be pointed out that self-energy corrections (such as GW) or electron–hole interactions (as described by the Bethe–Salpeter equation) are neglected. Self-energy corrections and electron–hole interactions are known to have a partially compensating effect on the band gap:^54^ while the former tends to increase the band gap, the electron–hole interactions make the optical band gap smaller. Due to this compensating effect, the present theoretical results can be seen as approximate spectra specifically showing the impact of the defects. However, quantitative differences between theory and experiment should be expected due to the neglect of many-body effects and also due to the difference in the dielectric environment. The theoretical spectra are shown in Figure 7. Absorption below the band gap (around 1.6 eV) is present for all defects and originates from transitions involving the defect states.

There is an optical transition for X-sub at 1.5 eV, which is in the same energy range of the D emission from the PL spectra between the valence band and an acceptor state of X-sub. This state results from the coupling of the conduction band at the *K-*point with the Cr defect state. The energy of this transition is similar to that of the MoSe_2_ ML with a vacancy.^55−58^

The weak transition involving a deep acceptor state at 0.9 eV is outside the spectral range of our experiments. We note that the coupling between the conduction band and the defect state shifts the conduction band minimum from *K* toward the Γ point (Supporting Figure S5). However, the resulting suppression of PL would not be visible in the experiment due to the low density of defects and only the local opening of the band gap.

Well-defined spin-degenerate acceptor levels are also introduced by Cr substituting the Mo atom in the lattice (Cr@Mo). Optical transitions from this defect state into the valence band states can be seen in Figure 7 in the range between 1.4 and 1.5 eV, which is also in a similar energy range to the D peak PL emission. The band-to-band transition is shifted to higher energy compared to the pristine MoSe_2_ ML because of the coupling between the conduction band and the defect state. However, similar to the X-sub configuration discussed above, it is unlikely to observe this blueshift in the PL spectra due to the low defect density.

The coupling of the defect and conduction band results in a gradual increase of the above-band-gap absorption for Cr substitution into the Se site (Cr@Se). This defect type also introduces a donor state at the Fermi level and two single-spin, deep defect levels. The signal from the donor state merges with the band-to-band absorption. Otherwise, the Cr defect at the Se site hardly affects the MoSe_2_ band structure. Several weak optical transitions are present at a large range of energies (down to 500 meV below the band gap).

Interstitial Cr introduces several deep defect levels in the band gap, and again the highest state couples to the conduction band shifting the conduction band minimum to the Λ point. The absorption spectrum does not contain discrete absorption lines but a gradually increasing absorption from 1.2 eV.

## 3 Discussion

Radiative recombination of an electron (e^–^) bound to a defect state with the valence band hole (h^+^) can explain the measured PL. Considering that our DFT calculations do not show donor states at high enough energy, the electron here is likely to occupy an acceptor. In this scenario, an exciton bound to a negatively charged acceptor (A^–^X) dissociates into A^–^h^+^ and a free electron in the conduction band. Following radiative recombination, A^–^h^+^ becomes neutral acceptor A^0^. Theoretical modeling of A^–^X indicated a binding energy of only a few meV compared with the A^0^ + e^–^ state,^10^ which is of the same order of magnitude as the activation energy of the D line determined from the Arrhenius plot. Among the potential defects identified by MD calculations, Cr@Mo, X-sub, and Cr@Se have nonzero matrix elements for optical transitions between acceptor states and valence band. Other configurations, e.g., interstitial Cr or Se vacancies, are unlikely to be present. Besides, neither would explain the data (see Figure 7 and Supporting Information Note 4).

The measured 1/*e* recombination time is longer than the lifetimes reported for band-to-band and localized state recombination in MoSe_2_. Low oscillator strength of the transition can result from the spatial separation of electrons and holes, as for Cr@Se or X-sub. However, since this lifetime is longer than that of the spin dark states in WSe_2_, which is only a few nanoseconds,^59^ this transition could also be from a spin-forbidden state. Such a state would correspond to the charge configuration of A^0^ for the Cr@Se defect in the absence of exchange interactions between electrons in the conduction band.

The *g*-factor of the D emission is negative but much smaller than those for X or X^–^. With large *g*-factors for electrons in the valence band, it implies either a large *g*-factor for an electron on the acceptor level near the conduction band (e.g., in Cr@Mo or X-sub configuration) and valley-selective transitions or reduced *g*-factor of holes in the valence band. The latter could be caused by the hybridization of the valence band with the defect level as in the Cr@Se configuration. Further insight would require higher magnetic field measurements and theoretical input.

## 4 Conclusions

In conclusion, we demonstrated the ultra-low-energy ion implantation of Cr ions (at 25 eV) into a MoSe_2_ ML. The implantation was performed with ions at 25 eV and an ion fluence of 3 × 10^12^ cm^–2^; the resulting material retains high optical quality as evidenced by clear excitonic PL. Implanted Cr ions introduce an additional low-energy PL signal at around 1.51 eV visible at the onset of n-doping. Molecular dynamics calculations identified defects that can be generated by implantation. We found that Cr atoms can substitute for both Mo and Se atoms. In the latter case, the Cr atom is slightly more likely to bind an additional Se atom than not. The defects’ stability, including interstitial Cr, depends on the post-implantation treatment and the final configuration of Se and Mo, which are not in the lattice. DFT calculations revealed that all of the probable defects introduce one or more defect states in the MoSe_2_ band gap with nonzero matrix elements for optical transitions. It is impossible to identify with certainty which defect is the origin of the D line, Cr at the Se site with the Se adatom (X-sub), and perhaps Cr at Mo (Cr@Mo) seems to fit best with the measured data. Further experiments, for example, the implantation of Cr only into the Se sublattice or implantation through the hBN protective layer to avoid environmental changes, could be considered to distinguish between the cases.

More generally, this study shows that implantation of heavier elements into the metal sublattice of TMD MLs is possible without the visible loss of material quality as evidenced by the unchanged excitonic PL of gated MoSe_2_. However, the implantation process is complex, and simulations of the possible outcomes are necessary to identify material systems of the desired properties. In the search for single photon emitting sites, it is also worth noting that upon implantation with a very low fluence, it should be possible to address individual Cr atoms at different lattice sites. Implantation of foreign atoms might be used to introduce catalytic sites in 2D materials or to add functionalization such as magnetism.

## 5 Methods

### 5.1 Atomistic Simulation

We used DFT MD as implemented in the VASP code.^60,61^ The Perdew–Burke–Ernzerhof (PBE) exchange and correlation functional was employed.^62^ The evolution of the system was modeled using the microcanonical ensemble. A cutoff value of 300 eV was chosen for DFT MD, and sampling over the Brillouin zone was done using a 3 × 3 × 1 *k*-point mesh. The time step was chosen to be 0.1 fs, which provided energy conservation better than 0.1 eV.

### 5.2 Band Structure and Absorption Spectral Calculation

Density functional theory (DFT) simulations were performed in supercells of 5 × 5 primitive unit cells. Each was constructed with lattice constants of *a* = 3.28 Å and *c* = 12.918 Å of the hexagonal lattice. An internal structure parameter of *z* = 0.125 was used. The defect systems were spatially relaxed using FLEUR^63,64^ until the residual atomic forces had fallen below 5 × 10^–2^ eV/Å. The subsequent calculation of the macroscopic dielectric function in SPEX^65,66^ is based on the random-phase approximation^67,68^ and includes local-field effects. Calculations of 2D materials with 3D periodic boundary conditions are computationally expensive because the decoupling of neighboring layers in the *z* direction requires large supercells in this direction. In the case of 2D systems with defects, the computational cost grows considerably, particularly in the case of low defect concentrations, because suppressing the unwanted defect–defect coupling requires large supercells in the *x* and *y* directions. To facilitate the calculations of the dielectric function, we had to reduce the reciprocal cutoff radius from 4.1 to 3.6 Bohr^–1^ in the case of the X-sub defect system. However, this should not affect the form of the respective spectrum shown in Figure 7.

The band structures presented in the Supporting Information are made up of 320 **k** points along the unfolded high-symmetry path Γ–*M*–*K*–Γ. Here, “unfolded” means that the high-symmetry points refer to those of the defect-free MoSe_2_ ML. The necessary unfolding of the band structures of the defect systems has been carried out with a new implementation^55^ in the FLEUR code, adapting the technique described in ref (69) to the LAPW basis.^70^ In this technique, a spectral weight is assigned to each state plotted in the band structure. The weight *w*_*n*_(**k**) for the *n*-th state at **k** of the unfolded path is given by
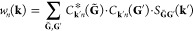
6where **k**′ = **k** + **G**″ with a suitable reciprocal lattice vector **G**″ that folds **k** back into the (smaller) Brillouin zone of the defect system. The **G**′ sum runs over the set of all reciprocal lattice vectors (of the defect system) at **k**′, and the **G̃** sum runs over the set of reciprocal lattice vectors (of the pristine system) at **k**. The latter is a subset of the former. The wave functions are represented in the LAPW basis {χ_**kG**_(**r**)} with coefficients *C*_**k***n*_(**G**) and overlap matrix *S*_**GG**′_(**k**) = ⟨χ_**kG**_|χ_**kG**′_⟩.^55^

### 5.3 Sample Preparation

Si with 90 nm thick dry-thermally grown SiO_2_ chips with 60 nm thick Ti/Au contacts (pre-patterned by electron beam lithography) were used as the substrate. Before flake transfer, the chips were cleaned in acetone and isopropanol (IPA) under bath sonication, blown dry with N_2_, and treated with oxygen plasma (300 W, 200 sccm for 10 min). Few-layer graphite, MoSe_2_ (from 2Dsemiconductors) MLs, and hBN (from Takashi Taniguchi and Kenji Watanabe) multilayers were mechanically exfoliated from bulk crystal using polydimethylsiloxane (PDMS) stamps (Gel-pak DGL X4 films) and transferred onto the substrate using the dry viscoelastic transfer process.^26^ The process was performed in a N_2_-filled glovebox. After transferring the graphite (5.5 nm thick) and bottom-hBN (20 nm thick) flakes, the sample was annealed in a H_2_/Ar (1:10 ratio) atmosphere at 300 °C for 3 h to improve the top surface for the subsequent MoSe_2_ ML transfer. After transferring the top-hBN (15 nm thick), the sample was annealed in low vacuum (5 × 10^–3^ mbar) at 150 °C for 2.5 h to improve interfaces in the vdW stack. Electrical contacts, provided by the Ti/Au lines, were made to the MoSe_2_ flake and the graphite backgate. After each transfer, the heterostructure surface was checked with atomic force microscopy to ensure a sufficiently flat area in the stack and to obtain the flakes’ thickness. MoSe_2_ ML’s quality was confirmed by Raman and PL spectroscopies at room temperature.^71^

### 5.4 Ion Implantation

Bronze tips were used to fix the sample on a holder, making contact with the sample’s Au pads and, thus, the ML. To remove volatile contamination from the sample, the sample chamber was then evacuated to 10^–9^ mbar for several hours. The sample was heated to 150 °C for 10 min to remove residual volatile adsorbates and then to 220 °C during the implantation. A foil was used as a feedstock to provide ^52^Cr^+^ ions. After extraction, the ions are decelerated from 30 keV to 25 eV directly in front of the sample. Since the deceleration voltage is set relative to the potential of the source anode, this energy represents the upper limit, with a tail toward lower energies. The fluence of the ions was set to 3 × 10^12^ cm^–2^. The fluence was verified by test implantations using the Rutherford backscatter spectrometry (more information in the Supporting Information Note 1). A detailed description of the source and the implantation system can be found in the references.^20,21^

### 5.5 Optical Measurements

PL spectroscopy was performed at 10 K (unless otherwise specified) in a He-cooled cold-finger cryostat (Cryoindustries) with a heating element (allowing a sample temperature range from 10 to 300 K). For PL measurements, the laser beam—688 nm (1.80 eV) from a Ti:Sa laser—is passed through a 680 ± 5 nm band-pass filter before being focused by an aspheric lens (NA = 0.47) into a spot of 1.6 μm in diameter on the sample. Unless otherwise specified, the laser power on the sample was at 1 μW for PL experiments. The PL signal is collected by the same lens and passed through a 700 nm low-pass filter before being focused by an achromatic doublet (NA = 0.24) through the entrance slit of a Czerny–Turner spectrometer, dispersed by a 600 l/mm grating onto a CCD camera. For gate dependence and temperature dependence PL, the laser power was kept at 1 μW. For PLE, the excitation power ranged from 2 to 5 μW, and the PL intensity is normalized to the power density for final data.

For time-resolved PL, the excitation was done using a pulsed laser at 660 nm (1.88 eV) with a 200 ps pulse length, a 2.5 MHz repetition rate, and a 2.8 μW average power. The PL signal is directed through an 800 nm (1.55 eV) low-pass filter on the detection path before entering an avalanche photodiode with a 30 ps time resolution. The histogram of the time difference between the laser pulses and PL emission was acquired with a time tagger.

The sample was mounted on an *x*–*y*–*z* Attocube stage in a He flow cryostat (attoDRY2100) for magneto-optics measurement at temperature *T* = 1.8 K. A magnetic field up to ±8 T was applied perpendicularly to the sample (Faraday configuration). The excitation laser beam (688 nm, i.e., 1.80 eV at 4 μW) was passed through a 680 nm band-pass filter and a linear polarizer in an H-configuration. The laser was focused by an aspheric lens (NA = 0.47) into a spot of ≈1.6 μm in diameter on the sample. The emitted PL was collected with the same objective. It was passed through a combination of λ/4, λ/2 waveplates, and a linear polarizer set to pass the σ^±^ polarized light. It was then propagated via a single-mode optical fiber toward the entrance slit of a Czerny–Turner spectrometer, where it was dispersed by a 600 l/mm grating onto a CCD camera. A long-pass filter (with a 700 nm band edge) was inserted between the fiber output and the spectrometer entrance to remove any remaining laser light.

## Data Availability

The data supporting the findings of this study are available within the paper and its Supporting Information files. Data are also available from the corresponding author upon reasonable request.

## Supporting Information Available

Additional experimental details on the RBS measurement of Cr implantation on the test ta-C substrate; spatial PL maps of D, X, and X^–^; polarization-resolved PL; Raman and PL spectroscopic studies of vacancies in MoSe_2_ ML; MD simulation and TEM studies of Cr implantation on MoS_2_ ML; unfolded band structure of Cr-implanted MoSe_2_ ML with various defect configurations; and charge carrier density conversion from gate voltage (PDF)

## Notes

All scripts used to generate the results in this study are available from the corresponding author upon reasonable request.
