# Supplementary material for: Optical Properties of MoSe2 Monolayer Implanted with Ultra-Low-Energy Cr Ions
Source: ACS Appl Mater Interfaces. 2023 Jul 11;15(29):35321–31. doi: 10.1021/acsami.3c05366 (PMC10375475; doi:10.1021/acsami.3c05366)
Supplement: Supplementary file 1 — am3c05366_si_001.pdf [file am3c05366_si_001.pdf]

Supporting Information
for
Optical properties of $MoSe_2$ monolayer implanted with ultra-low energy Cr ions

Minh N. Bui,[1,2,*] Stefan Rost,[3,2] Manuel Auge,[4] Lanqing Zhou,[1,2] Christoph Friedrich,[3] Stefan Blügel,[3,2] Silvan Kretschmer,[5] Arkady V. Krasheninnikov,[5,6] Kenji Watanabe,[7] Takashi Taniguchi,[8] Hans C. Hofsäss,[4] Detlev Grützmacher,[1,2] and Beata E. Kardynał[1,2,†]

[1] *Peter Grünberg Institute 9 (PGI-9), Forschungszentrum Jülich, 52425 Jülich, Germany*
[2] *Department of Physics, RWTH Aachen University, 52074 Aachen, Germany*
[3] *Peter Grünberg Institute 1 (PGI-1) and Institute for Advanced Simulation 1 (IAS-1), Forschungszentrum Jülich and JARA, 52425 Jülich, Germany*
[4] *II. Institute of Physics, University of Göttingen, 37077 Göttingen, Germany*
[5] *Institute of Ion Beam Physics and Materials Research, Helmholtz-Zentrum Dresden-Rossendorf, 01328 Dresden, Germany*
[6] *Department of Applied Physics, Aalto University, P.O.Box 11100, 00076 Aalto, Finland*
[7] *Research Center for Functional Materials, National Institute for Materials Science, 1-1 Namiki, Tsukuba 305-0044, Japan*
[8] *International Center for Materials Nanoarchitectonics, National Institute for Materials Science, 1-1 Namiki, Tsukuba 305-0044, Japan*

Contents

Supplementary note 1 - Rutherford backscattering spectrometry S-2

Supplementary note 2 - Spatial distribution of D peak S-3

Supplementary note 3 - Polarization resolved PL S-4

Supplementary note 4 - Raman and PL spectroscopy studies of vacancies in $MoSe_2$ ML S-5

Supplementary note 5 - MD simulation and TEM studies of Cr implantation in MoS_2 S-7

Supplementary note 6 - Unfolded band structures including implanted defects S-8

Supplementary note 7 - Charge carrier density conversion from gate voltage S-9

References S-9

* m.bui@fz-juelich.de
† b.kardynal@fz-juelich.de

Supplementary note 1 - Rutherford backscattering spectrometry

Rutherford backscattering spectrometry (RBS) studies were carried out to verify the nominal implantation fluence of our experiments. An 860 keV helium ion beam was generated using a Cockcroft-Walton accelerator and then directed onto the sample surface. The backscattered ions were detected by a silicon charged-particle radiation detector under an angle of 165° to the ion beam direction. The semiconductor detector has an active area of 25 mm^2 and is located at a distance of 88 mm. Figure S1 shows an RBS spectrum for Cr implantation at 25 eV with a fluence of 3×10^{15} cm^{-2} on a Si/ta-C substrate. Fitting procedure using SIMNRA [1] gives Cr density of $3.03 \pm 0.13 \times 10^{15}$ cm^{-2}, which agrees well with the given values in the Methods section. The test implantations are performed with a fluence of 3×10^{15} cm^{-2} to achieve a high count rate in RBS in a reasonable acquisition time and to perform a better fit in SIMNRA.

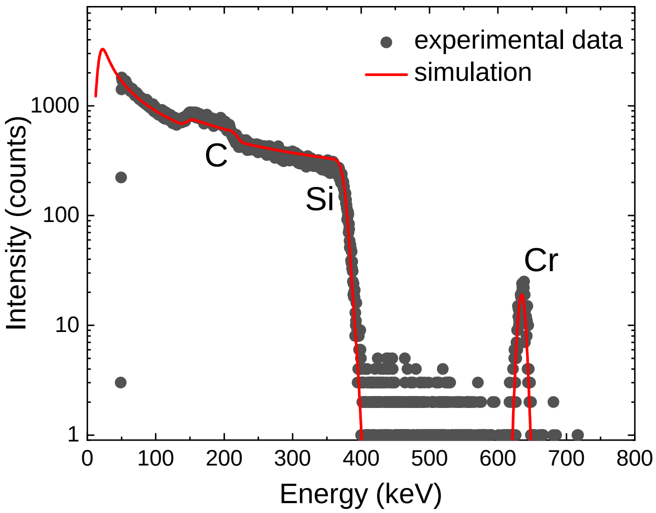

Figure S1: RBS spectrum for Se implanted in ta-C on a Si substrate. The implantation was performed at 25 eV with a fluence of 3×10^{15} cm^{-2}. Fit curve was done using SIMNRA.

Supplementary note 2 - Spatial distribution of D peak

Figure S2 shows the 2D integrated micro-PL (µ-PL) maps for D, X and X^- emissions. Compared to the excitonic emission map of X and X^-, D emission is observed only in the implanted $MoSe_2$ ML area and does not come from the hBN or Gr layer underneath. The emission is also not from localised sites, e.g. wrinkles, scratches or bubbles in the ML, but rather the whole ML. The apparent difference between the bright and dark halves in the ML is likely from inhomogeneous doping provided by the bottom Gr gate.

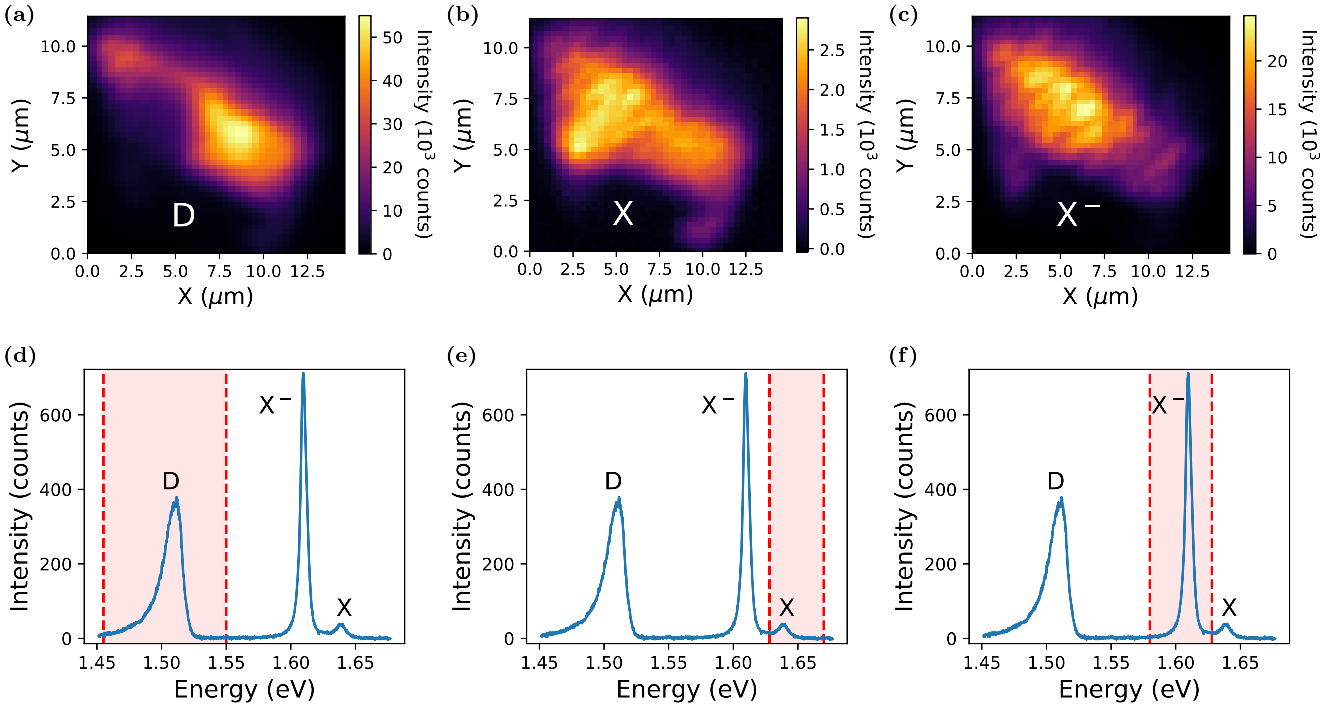

Figure S2: µPL maps of Cr-implanted $MoSe_2$ ML. The PL intensity was integrated around (a) D, (b) X, and (c) X^- emission. The integrated PL signal is summed over the energy range indicated in the shaded region in respective single spectra in (d), (e), and (f).

Supplementary note 3 - Polarization resolved PL

A linear polariser, a $\lambda/2$-waveplate and a $\lambda/4$-waveplate were inserted in the excitation path (before the beam-splitter) to set the polarisation state of the laser beam from a diode laser (655 nm in wavelength and 4.59 µW in power on the sample). On the detection path, a $\lambda/4$-waveplate, a $\lambda/2$-waveplate and a linear polariser were placed to set the detected polarisation state. D line shares polarisation properties with the X and X^- as shown in figure S3. Excitation with circularly polarised light resulted in a low degree of circular dichroism for X and X^- emissions, typical for $MoSe_2$ [2–4]. The degree of dichroism, defined as $P_C = (I_+ - I_-)/(I_+ + I_-)$ where $I_\pm$ are PL intensity detected in $\sigma_\pm$ states, is similar for D compared with X and X^-.

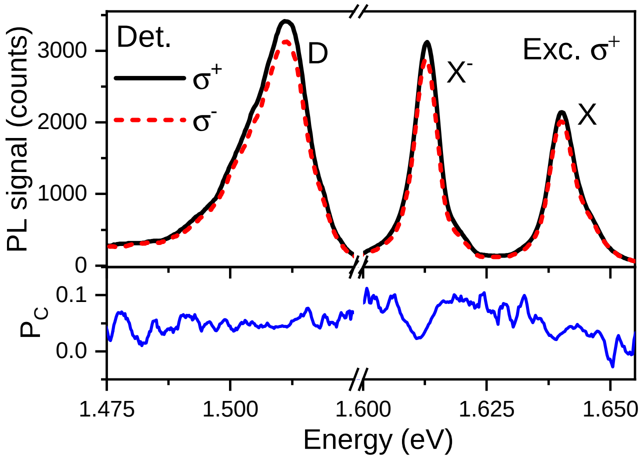

Figure S3: Polarization-dependent PL of Cr-implanted $MoSe_2$ at 10 K. The sample is excited with left (σ^+) circularly polarised light, and PL is detected in left - red curve - and right (σ^-) - dotted black curve - states. D, X^- and X exhibit low circular dichroism P_C at below 0.05.

Supplementary note 4 - Raman and PL spectroscopy studies of vacancies in $MoSe_2$ ML

According to MD simulation (table 1 in main text), we should exclude the Se vacancy as the signal's origin. Vacancy introduces a donor state at the Fermi level hybridised with the valence band and an acceptor state deep in the bandgap [5–8]. An optical transition between the defect levels is not allowed (figure 7).The lowest allowed energy optical transition is between the valence band state at the Γ point and the deep acceptor level. The existing literature reports that vacancies form non-radiative recombination sites that quench PL [9, 10] or that they contribute to sub-bandgap PL but only at low temperature [11–13], which is in contrast with our data (see RT PL from our samples in figure S4b). In addition, the energy shift of the D line with the temperature [14] (figure 4c) is weaker than that of X, unlike the fast change reported for vacancies in $MoSe_2$ [11]. At sufficient density (around 8%), vacancies can blueshift the PL and downshift the Raman lines [7]. In contrast, we did not see such changes in our Cr-implanted $MoSe_2$ ML (figure S4).

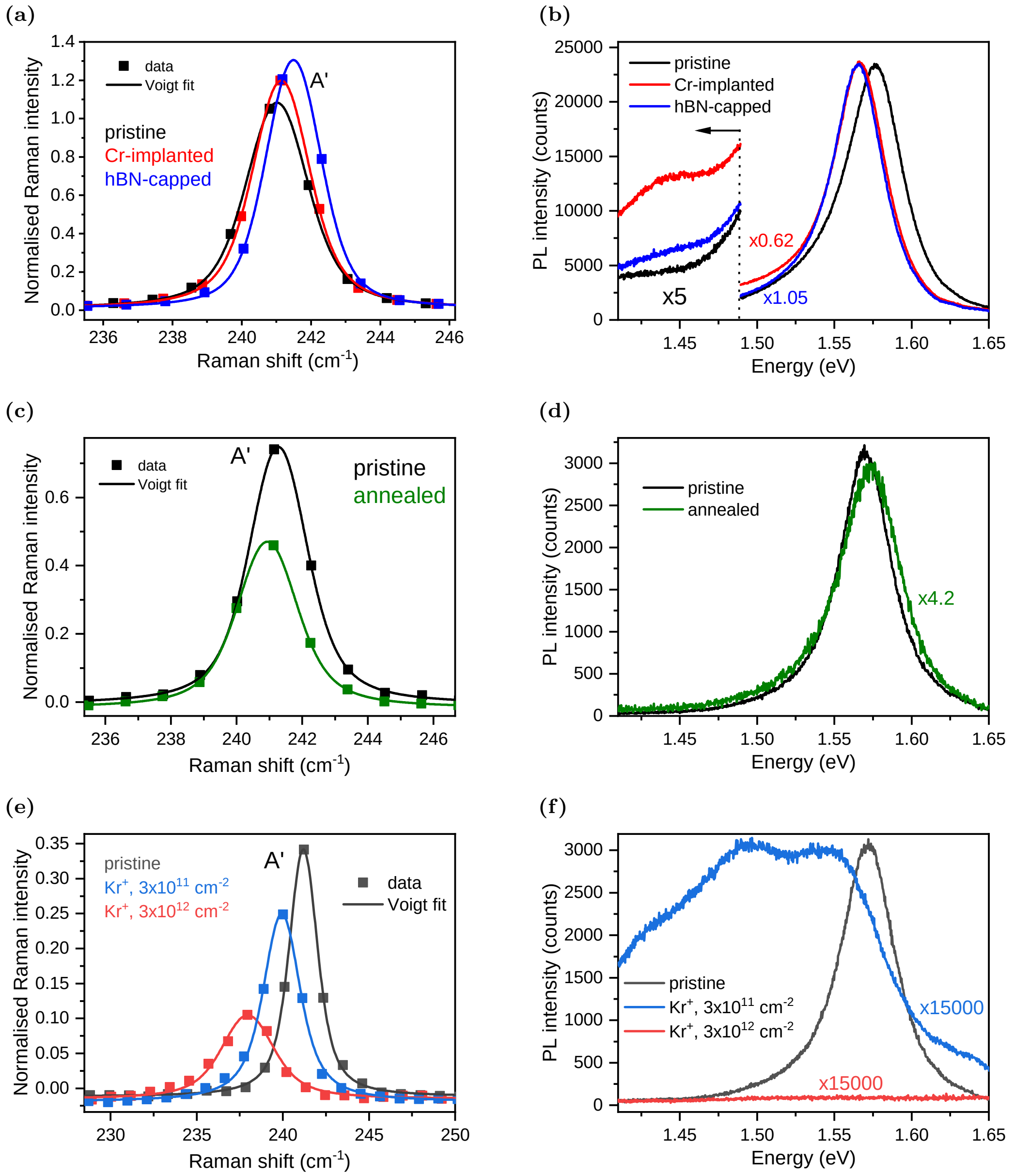

Figure S4: Optical spectroscopy of defected $MoSe_2$ ML. Room temperature Raman (a,c,e) and PL (b,d,f) spectra of Cr-implanted $MoSe_2$ ML (a,b), annealed ML (c,d) and Kr-implanted MLs (e,f), with a pristine ML's spectrum for comparison. Raman spectra are all normalised to Si signal at 520.5 cm^{-1}.

To ensure that one can exclude the role of vacancies in the D emission, room temperature Raman and PL spectra

of $MoSe_2$ MLs with vacancies were compared to Cr-implanted $MoSe_2$. To create vacancies, one sample of $MoSe_2$ was annealed at 300 °C in low vacuum (5×10^{-2} mbar) for 2.5 hours [13]. Another two samples were implanted with Kr^+, which, together with other noble gases, is commonly used for creating vacancies and their complexes in 2D materials [12, 13, 15–22]. Ion implantation was done at 25 eV energy for introducing vacancies [23, 24], with fluences of 3×10^{11} and 3×10^{12} cm^{-2} at elevated temperature of 220 °C, after being pre-annealed for 30 minutes at the same temperature in the implant chamber to remove volatile adsorbates. The higher fluence corresponds to the same Cr fluence in the main experiment. The lower fluence is about 10 times higher than the upper limit of potential vacancies density predicted for the Cr implanted sample by the atomistic MD simulation in the main text.

Figure S4 shows the room temperature Raman and PL spectra of $MoSe_2$ MLs before and after Cr implantation, annealing and Kr implantation (as already described above or in the main text). Since slight variations in PL and Raman spectra are possible from sample to sample, the spectra from the same ML before and after processing are shown. Cr-implantation introduces a small upshift in the out-of-plane Raman vibrational mode A' (figure S4a). The upshift is expected for Cr atoms in the lattice because it stiffens the lattice bonds and increases the restoring force and the A' frequency. Annealing and Kr implantation downshift this Raman line (figures S4c and S4e). Such a downshift has been reported before for $MoSe_2$ MLs with vacancies [7, 25–27]. It was explained by the lattice bond loosening and lowering the restoring force. PL emission of the Cr-implanted ML is 15 meV redshifted compared with the pristine ML. It also shows an emission band around 120 meV below the free exciton line (figure S4b). This low-energy emission is also observed at low temperatures. On the other hand, the annealed ML has a very slight blueshift (figure S4d), consistent with several other reports [7, 25]. Kr-implanted MLs' PL is heavily quenched (figure S4f). Some signal is visible only when the excitation laser power is raised 15000 times (from 17 nW to 261 μW). The disagreement from these experiments doesn't favour vacancies being the origin of the D emission from the Cr-implanted ML.

Raman and PL spectra presented in this section were acquired under ambient conditions (room temperature and pressure) using a confocal Raman microscope (Renishaw inVia) with 532 nm excitation laser (Coherent Compass 315M 150SL). The laser power was set between 0.017 and 261 μW for sufficient signal intensity while preventing the sample from heating during exposure. An objective lens (50×, NA = 0.75, Leica N-plan EPI) collected the Raman signal, which was then dispersed by a 2400 l/mm grating on the CCD camera, giving a spectral resolution of 1 cm^{-1}. PL signal was dispersed with a 600 l/mm grating, yielding a resolution of 0.147 nm.

Supplementary note 5 - MD simulation and TEM studies of Cr implantation in MoS_2

To compare the types of defects obtained in the simulations to the available experimental data, we also modelled ion implantation into ML MoS_2. The results are presented in supplementary table S1. We note that interstitials cannot exist in ML MoS_2 due to a smaller unit cell size than that of $MoSe_2$. Configurations predicted by the simulation were observed in the atomic-resolution TEM characterisation of ML MoS_2 implanted with Cr under similar conditions [28]. It was noted that the relative abundance of defects, determined by the intensity-based atomic site assignment, was heavily affected by the presence of carbon contamination on the ML surface. In addition to the poor resolution in the experimental images, this renders accurate quantification of the elements difficult.

Table S1: Results of DFT MD simulations of 25 eV Cr ion irradiation on single layer MoS_2. The probabilities p of likely defect configurations to appear along with the formation energies E_f of these configurations are listed.

	p	E_f [eV]
adatom	0.12	-0.98
X-sub	0.12	-0.74
interstitial	0.00	0.93
Cr@Mo	0.41	3.06
Cr@S	0.12	2.87
V_S	0.13	6.53
passed through	0.10	0.00

Supplementary note 6 - Unfolded band structures including implanted defects

The main text shows the dielectric functions of $MoSe_2$ MLs containing several Cr-implantation introduced defects. In the following, their unfolded band structures are shown, which means that the band structures are shown in the first Brillouin zone of the pristine unit cell to visualise better the perturbation that the defect introduces. The methodology is briefly recapitulated in the method section of the main text.

Single atomic defects should show up as flat energy levels if the computational supercell is large enough for the periodic images of the defect atom not to interact. It is not always the case here due to the computational resources that such large supercell would require. Wherever a defect state crosses and interacts with a band of the host lattice, a hybridisation of the bands is visible.

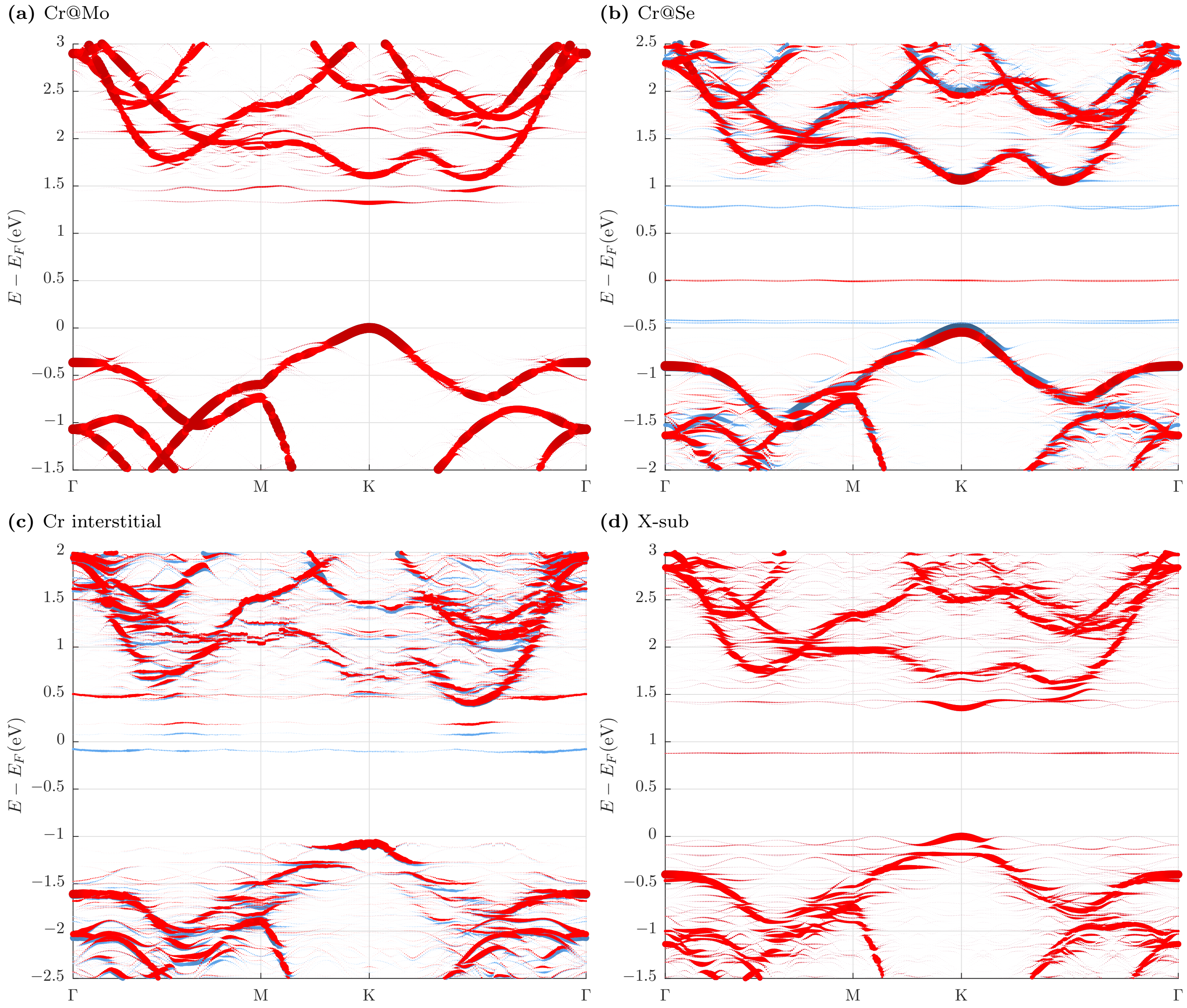

Figure S5: DFT calculation for unfolded band structure of Cr-implanted $MoSe_2$ ML with different Cr defect configurations: (a) Cr@Mo, (b) Cr@Se, (c) interstitial Cr atom, (d) X-sub. Calculations were performed in 5×5 supercells (with a reciprocal cutoff radius of 4.1 Bohr^{-1} for (d), and 3.6 Bohr^{-1} for its respective absorption spectrum in the main text) [5]. The calculations include two spins, shown here in red and blue, without spin-orbit coupling. Defects (and bands) in (a) and (d) are spin degenerate. Defect-induced states in (b) and (c) can be occupied by one electron. The influence of the implanted defect is visible via the defect-induced states inside the bandgap. A thick symbol size (high unfolding weight) means that the symmetry of this state corresponds to the one of the primitive cell (pristine material).

Supplementary note 7 - Charge carrier density conversion from gate voltage

The carrier concentration n is estimated by the simple parallel plate capacitor model [29, 30]. The bottom hBN flake acts as a dielectric of the capacitor with dielectric constant $\epsilon_{\mathrm{hBN}} \approx 3.4$ [30–32]. Upon applying gate voltage V_g, we have

$$C = \frac{Q}{(V_g - V_0)} = \epsilon_{\mathrm{hBN}}\epsilon_0 \frac{A}{t}$$

with C the capacitance of the graphite/hBN/$MoSe_2$ stack, Q the charge on $MoSe_2$ ML at V_g, V_0 being the gate voltage at charge neutrality point (taken to be at minimum trions PL intensity), ϵ_0 the vacuum permittivity, A the hBN flake's area, and the flake's thickness $t = 20$ nm. Rearranging the above equation gives the charge carrier density n on the ML

$$n = \frac{Q}{eA} = \epsilon_{\mathrm{hBN}}\epsilon_0 \frac{(V_g - V_0)}{et}$$

where $e = \text{-}1.602 \times 10^{-19}$ C is the electron charge.

[1] Mayer, M. SIMNRA User's Guide. Report IPP 9/113, 1997; `https://home.mpcdf.mpg.de/~mam/Report%20IPP%209-113.pdf`, accessed at 10:33 12.04.2023.

[2] MacNeill, D.; Heikes, C.; Mak, K. F.; Anderson, Z.; Kormányos, A.; Zólyomi, V.; Park, J.; Ralph, D. C. Breaking of Valley Degeneracy by Magnetic Field in Monolayer $MoSe_2$. *Phys. Rev. Lett.* **2015**, *114*, 037401.

[3] Kioseoglou, G.; Hanbicki, A. T.; Currie, M.; Friedman, A. L.; Jonker, B. T. Optical polarization and intervalley scattering in single layers of MoS_2 and $MoSe_2$. *Scientific Reports* **2016**, *6*, 25041.

[4] Wang, G.; Palleau, E.; Amand, T.; Tongay, S.; Marie, X.; Urbaszek, B. Polarization and time-resolved photoluminescence spectroscopy of excitons in $MoSe_2$ monolayers. *Applied Physics Letters* **2015**, *106*, 112101.

[5] Rost, S. H. Computational study of structural and optical properties of two-dimensional transition-metal dichalcogenides with implanted defects. Dissertation, RWTH Aachen University, Jülich, 2023.

[6] Iberi, V.; Liang, L.; Ievlev, A. V.; Stanford, M. G.; Lin, M.-W.; Li, X.; Mahjouri-Samani, M.; Jesse, S.; Sumpter, B. G.; Kalinin, S. V.; Joy, D. C.; Xiao, K.; Belianinov, A.; Ovchinnikova, O. S. Nanoforging Single Layer $MoSe_2$ Through Defect Engineering with Focused Helium Ion Beams. *Scientific Reports* **2016**, *6*, 30481.

[7] Mahjouri-Samani, M. et al. Tailoring Vacancies Far Beyond Intrinsic Levels Changes the Carrier Type and Optical Response in Monolayer $MoSe_{2-x}$ Crystals. *Nano Letters* **2016**, *16*, 5213–5220, PMID: 27416103.

[8] Shafqat, A.; Iqbal, T.; Majid, A. A DFT study of intrinsic point defects in monolayer $MoSe_2$. *AIP Advances* **2017**, *7*, 105306.

[9] Tanoh, A. O. A.; Alexander-Webber, J.; Fan, Y.; Gauriot, N.; Xiao, J.; Pandya, R.; Li, Z.; Hofmann, S.; Rao, A. Giant photoluminescence enhancement in $MoSe_2$ monolayers treated with oleic acid ligands. *Nanoscale Adv.* **2021**, *3*, 4216–4225.

[10] Amani, M. et al. Near-unity photoluminescence quantum yield in MoS_2. *Science* **2015**, *350*, 1065–1068.

[11] Li, X.; Puretzky, A. A.; Sang, X.; KC, S.; Tian, M.; Ceballos, F.; Mahjouri-Samani, M.; Wang, K.; Unocic, R. R.; Zhao, H.; Duscher, G.; Cooper, V. R.; Rouleau, C. M.; Geohegan, D. B.; Xiao, K. Suppression of Defects and Deep Levels Using Isoelectronic Tungsten Substitution in Monolayer $MoSe_2$. *Advanced Functional Materials* **2017**, *27*, 1603850.

[12] Klein, J. et al. Site-selectively generated photon emitters in monolayer MoS_2 via local helium ion irradiation. *Nat. Commun.* **2019**, *10*, 2755.

[13] Mitterreiter, E. et al. The role of chalcogen vacancies for atomic defect emission in MoS_2. *Nat. Commun.* **2021**, *12*, 3822.

[14] O'Donnell, K. P.; Chen, X. Temperature dependence of semiconductor band gaps. *Applied Physics Letters* **1991**, *58*, 2924–2926.

[15] Lucchese, M.; Stavale, F.; Ferreira, E. M.; Vilani, C.; Moutinho, M.; Capaz, R. B.; Achete, C.; Jorio, A. Quantifying ion-induced defects and Raman relaxation length in graphene. *Carbon* **2010**, *48*, 1592–1597.

[16] Lehtinen, O.; Kotakoski, J.; Krasheninnikov, A. V.; Tolvanen, A.; Nordlund, K.; Keinonen, J. Effects of ion bombardment on a two-dimensional target: Atomistic simulations of graphene irradiation. *Phys. Rev. B* **2010**, *81*, 153401.

[17] Junge, F.; Auge, M.; Zarkua, Z.; Hofsäss, H. Lateral Controlled Doping and Defect Engineering of Graphene by Ultra-Low-Energy Ion Implantation. *Nanomaterials* **2023**, *13*.

[18] Kianinia, M.; White, S.; Fröch, J. E.; Bradac, C.; Aharonovich, I. Generation of Spin Defects in Hexagonal Boron Nitride. *ACS Photonics* **2020**, *7*, 2147–2152.

[19] Shi, W.; Lin, M.-L.; Tan, Q.-H.; Qiao, X.-F.; Zhang, J.; Tan, P.-H. Raman and photoluminescence spectra of two-dimensional nanocrystallites of monolayer WS_2 and WSe_2. *2D Materials* **2016**, *3*, 025016.

[20] Murray, R.; Haynes, K.; Zhao, X.; Perry, S.; Hatem, C.; Jones, K. The Effect of Low Energy Ion Implantation on MoS_2. *ECS Journal of Solid State Science and Technology* **2016**, *5*, Q3050.

[21] Maguire, P.; Fox, D. S.; Zhou, Y.; Wang, Q.; O'Brien, M.; Jadwiszczak, J.; Cullen, C. P.; McManus, J.; Bateman, S.; McEvoy, N.; Duesberg, G. S.; Zhang, H. Defect sizing, separation, and substrate effects in ion-irradiated monolayer two-dimensional materials. *Phys. Rev. B* **2018**, *98*, 134109.

[22] Klein, J. et al. Engineering the Luminescence and Generation of Individual Defect Emitters in Atomically Thin MoS_2. *ACS Photonics* **2021**, *8*, 669–677.

[23] Ghorbani-Asl, M.; Kretschmer, S.; Spearot, D. E.; Krasheninnikov, A. V. Two-dimensional MoS_2 under ion irradiation: from controlled defect production to electronic structure engineering. *2D Materials* **2017**, *4*, 025078.

[24] Ghaderzadeh, S.; Ladygin, V.; Ghorbani-Asl, M.; Hlawacek, G.; Schleberger, M.; Krasheninnikov, A. V. Freestanding and Supported MoS_2 Monolayers under Cluster Irradiation: Insights from Molecular Dynamics Simulations. *ACS Applied Materials & Interfaces* **2020**, *12*, 37454–37463, PMID: 32814400.

[25] Azam, N.; Boebinger, M. G.; Jaiswal, S.; Unocic, R. R.; Fathi-Hafshejani, P.; Mahjouri-Samani, M. Laser-Assisted Synthesis of Monolayer 2D $MoSe_2$ Crystals with Tunable Vacancy Concentrations: Implications for Gas and Biosensing. *ACS Applied Nano Materials* **2022**, *5*, 9129–9139.

[26] Kim, H. J.; Van Quang, N.; Nguyen, T. H.; Kim, S.; Lee, Y.; Lee, I. H.; Cho, S.; Seong, M.-J.; Kim, K.; Chang, Y. J. Tuning of Thermoelectric Properties of $MoSe_2$ Thin Films Under Helium Ion Irradiation. *Nanoscale Research Letters* **2022**, *17*, 26.

[27] Xiao, D.; Ruan, Q.; Bao, D.-L.; Luo, Y.; Huang, C.; Tang, S.; Shen, J.; Cheng, C.; Chu, P. K. Effects of Ion Energy and Density on the Plasma Etching-Induced Surface Area, Edge Electrical Field, and Multivacancies in $MoSe_2$ Nanosheets for Enhancement of the Hydrogen Evolution Reaction. *Small* **2020**, *16*, 2001470.

[28] Hennessy, M.; O'Connell, E. N.; Auge, M.; Moynihan, E.; Hofsäss, H.; Bangert, U. Quantification of Ion-Implanted Single-Atom Dopants in Monolayer MoS_2 via HAADF STEM Using the TEMUL Toolkit. *Microscopy and Microanalysis* **2022**, *28*, 1407–1416.

[29] Robert, C. et al. Spin/valley pumping of resident electrons in WSe_2 and WS_2 monolayers. *Nature Communications* **2021**, *12*, 5455.

[30] Li, M.; Biswas, S.; Hail, C. U.; Atwater, H. A. Refractive Index Modulation in Monolayer Molybdenum Diselenide. *Nano Letters* **2021**, *21*, 7602–7608, PMID: 34468150.

[31] Pierret, A. et al. Dielectric permittivity, conductivity and breakdown field of hexagonal boron nitride. *Materials Research Express* **2022**, *9*, 065901.

[32] Laturia, M. L., Akashand Van de Put; Vandenberghe, W. G. Dielectric properties of hexagonal boron nitride and transition metal dichalcogenides: from monolayer to bulk. *npj 2D Materials and Applications* **2018**, *2*, 6.
